# Effect of Shot Peening on the Mechanical Properties and Cytotoxicity Behaviour of Titanium Implants Produced by 3D Printing Technology

**DOI:** 10.1155/2019/8169538

**Published:** 2019-12-19

**Authors:** Remigiusz Żebrowski, Mariusz Walczak, Agnieszka Korga, Magdalena Iwan, Mirosław Szala

**Affiliations:** ^1^Department of General Surgery Center of Oncology of the Lublin Region St. John of Dukla, Dr. K. Jaczewskiego St. 7, 20-090 Lublin, Poland; ^2^Department of Materials Engineering, Faculty of Mechanical Engineering, Lublin University of Technology, Nadbystrzycka St. 36, 20-618 Lublin, Poland; ^3^Independent Medical Biology Unit, Medical University of Lublin, Chodzki 6, 20-093 Lublin, Poland; ^4^Independent Medical Biology Unit, Medical University of Lublin, Chodzki 6, 20-093 Lublin, Poland; ^5^Department of Materials Engineering, Faculty of Mechanical Engineering, Lublin University of Technology, Nadbystrzycka St. 36, 20-618 Lublin, Poland

## Abstract

Structural discontinuities characterize the implants produced directly from metal powders in 3D printing technology. Mainly, the surface defects should be subjected to procedures associated with surface layer modification (likewise shot peening) resulting in the increase of the implant service life maintaining optimal biocompatibility. Therefore, the purpose of the present study was to investigate the effect of type of shot used for the peening process on the Ti-6Al-4V implants functional properties as well as the biological properties. The components were produced by DMLS (direct metal laser sintering) additive technology. The surfaces of titanium specimens have been subjected to the shot peening process by means of three different shots, i.e., CrNi steel shot, crushed nut shells, and ceramic balls shot. Then, the specimens have been subjected to profilometric analysis, microhardness tests, and static strength testing as well as to the assessment of biocompatibility in respect of cytotoxicity using human BJ fibroblasts. The shot peening process causes the strengthening of surface layer and the increase of strength parameters. Furthermore, the test results indicate good biocompatibility of surfaces being tested, and the effect of shot peening process on the titanium alloy cytotoxicity is acceptable. At the same time, most favourable behaviour in respect of cytotoxicity has been found in the case of surfaces modified by means of ceramic balls > nut shells > CrNi steel shot correspondingly.

## 1. Introduction

Additive manufacturing in healthcare sector is still in progress [1]. Nevertheless, it is already applied in in numerous different ways in many fields of medicine and becoming increasingly popular in particular when it is required to produce an implant with complex shapes adapted for anatomic conditions of the patient. Furthermore, additive technologies work very well in the case of the necessity to produce porous or cellular structures with proper mechanical strength and rigidity of implants when their production is impossible by means of conventional technologies, e.g., casting, plastic forming, or material removal processing [2, 3].

The correct selection of material depends on the type of 3D printing technology as well as it is directly associated with the requirements of implant model itself. In connection with medical applications, diversified mechanical properties of materials are required for various anatomic structures and, consequently, it is required to use various range of materials in order to meet high requirements in the scope of object durability as well as biocompatibility [4]. The medical products made of titanium seem to have already an established position among known metal biomaterials, in particular in the field of orthopaedics and in dental prosthetics due to excellent strength and corrosion parameters as well as their acceptance by living tissues. At the moment, additive technologies belong to the most promising production methods of metal implants made of Ti-6Al-4V [5, 6]. It has been observed [2, 3, 6, 7] that the products obtained by means of 3D printing technology, even in the case of consideration of optimal parameters of printing technology recommended by the manufacturers of metal powders laser sintering systems, are characterized by certain structural discontinuities (defects) in surface layer. This type of implant surface defects in the form of unmelted metal powder grains or pores occurred as a result of collapse of welding puddle may reduce useful parameters of implants and, in consequence, may lead to the necessity to carry out revision surgeries. Despite the fact that the literature of the subject signalizes the problem of proper surface finish of 3D-manufactured biomedical components, the solution has not been presented. Thus, the shot peening technology seems to be promising.

Moreover, the direct metal powder laser sintering (DMLS) additive technology itself causes the residual stresses in the product to occur [6]. Therefore, for this type of product, it is favourable to carry out the shot peening process that decreases stresses and causes the increase of metal elements strength as a result of reinforcement of the surface layer durability. However, as a result of the surface shot peening process, shot grains penetrate (are driven) into the superficial layer, except for the improvement of mechanical properties may slightly deteriorate the corrosion resistance of the products being modified and may contribute to the release of elements ions into surrounding tissues and to cytotoxicity [7, 8]. Therefore, the local unfavorable reactions of tissue are associated with elements being released mainly due to the fact that the local tissue is exposed to significantly higher concentrations of released metallic ions (i.e., metallosis effect). Furthermore, the degree of cytotoxicity and consequently the biocompatibility of metallic alloys are associated with the metal alloy composition as well as with elements released from the alloy to surrounding environment (nutrient medium) or body tissues. In [5, 9, 10], it has been indicated that biocompatibility of an implant depends, to a large extent, on the roughness and morphology of the surface. Substantial majority of material failures in implants, including strain cracks and abrasive or corrosive wearing, are initiated on the product surface. Recently, many scientific publications were concentrated on the solution of this problem. The quality of surface layer completion and biocompatibility are the key factors influencing the effective implantation of prostheses. However, the literature does not report how the shot peening process parameters (e.g., shot type or properties) influence the biocompatibility of a peened metal surface.

Therefore, the purpose of the article is to determine the most accurate shot peening process parameters for mechanical properties and cytotoxicity of the implants made of Ti-6Al-4V alloy by means of DMLS (direct metal laser sintering) additive technology. To finish the surface of the 3D-printed titanium components, the three processes varying with shot peening materials, i.e., CrNi steel shot, crushed nutshells, and ceramic shot, have been investigated. Finishing the surface layer with the usage of shot peening treatment seems beneficial especially for improving the functional properties of additive manufactured metal biomaterials. On the other hand, the literature survey indicates that there is no information about how the shot peening influences the personalized titanium implants manufactured with DMLS technology. Especially the application of nutshell granules as a shot material, used for production the durable and biocompatible components, is a new attempt previously not reported by the literature. This study is considered to be valuable from a scientific point of view as well the results can be useful in practice.

## 2. Materials and Methods

### 2.1. Specimen Preparation and Postsintering Treatments

Gas-atomized Ti-6Al-4V alloy powder has been used for specimen production. The specimens have been printed by means of DMLS (direct metal laser sintering) technique using the EOSINT M280 metal powder laser sintering system (EOS GmbH, Germany). Among others, the essential printing parameters are as follows: distance between the paths of 0.1 mm, laser beam speed of 1250 mm/s, the thickness of melted powder layers of 30 *μ*m, and applied power of laser beam of 170 W. After the sintering process, the specimens have been subjected in the chamber furnace N41/N (Nabertherm, Germany) to postsintering heat treatments stress relieving in vacuum during the period of 4 h at temperature of 800°C and to cooling in argon thereafter using process rinsing rate of 25 l/min.

Then, the specimen surfaces have been subjected to the shot peening process on the Peenmatic micro 750S device (IEPCO, Switzerland) until a coverage area of 100% is achieved using working pressure of 0.4 MPa. The shot peening process was carried out perpendicularly to the surface, while the distance between the nozzle and the face of surface being processed was equal to ∼25 mm. Three different media have been used, i.e., CrNi steel shot, crushed nut shells and ceramic balls. Principal parameters of the materials used in the shot peening process are included in Table 1. The condition of surface layer after shot peening process has been subjected to analysis on a Contour GT optical profilometer (Bruker, Germany). The measurements were carried out under magnification 5.5X. The profilometric analysis encompassed a surface area of 5 mm × 5 mm using the VSI method (Vertical Scanning Interferometry), and the obtained signals have been converted by means of BrukerVision64 software. The depth of the reinforced layer has been evaluated on metallographic cross-sections by means of the Eclipse MA200 microscope (Nikon, Japan).

### 2.2. Mechanical Tests

The measurements of microhardness of modified surfaces (outer faces of specimen in the *X*-*Y* horizontal plane) were carried out at the load of 0.2 kg (which corresponds to HV0.2) by means Vicker's FM-700 microhardness tester with the ARS 900 automatic system (Future-Tech Corp., Japan). The indentation dwell time was equal to 10 s. The specimens for microhardness tests have been produced in the form of discs with a diameter of 12 mm and thickness of 3 mm. Thirty indentations have been completed for each group of specimens.

**Figure 1:**
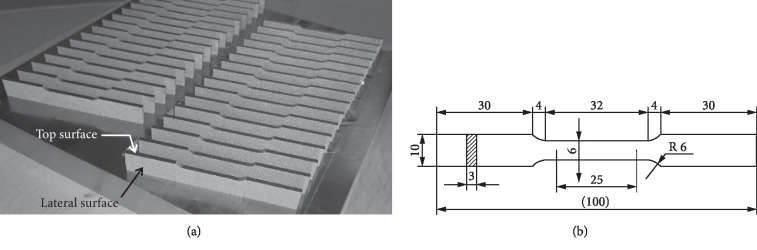
Specimens according to ASTM E-8 specification for tensile test (a); dimensions (in mm) of tensile specimens (b).

Six specimens for tensile strength testing have been prepared for each series, and their size and shape were in conformation with the ASTM E-8 standard (Figure 1). After the specimens were cut off from the die, one adhering side was subjected to machining on CNC Haas UMC750 milling centre and all the surfaces were subjected to the shot peening process. Strength properties have been tested by means of Z100 universal testing machine (Zwick, Germany) equipped with measuring head 50 kN and with the Makro 205 extensometer. Testing speed (traverse feed) was equal to 20 mm/min.

### 2.3. Biological Tests

#### 2.3.1. Cell Culture and Treatment

Biocompatibility evaluation in terms of cytotoxicity was conducted in accordance with ISO 10993-5:2009 using the extract test and direct contact test. Titanium alloy specimens (as in the case of the microhardness tests) have been produced in the form of discs with a diameter of 12 mm and thickness of 3 mm. Thirty indentations have been completed for each group of specimens.

In the study, human skin fibroblast BJ (ATCC, USA) cell line was used. The cells were cultured under standard conditions: at 37°C, in 5% CO_2_ atmosphere, in EMEM medium, respectively (USA, ATCC) supplemented with 10% foetal bovine serum (USA, ATCC). Prior to the experiments, all cell lines have been tested for the presence of mycoplasma using the LookOut® Mycoplasma PCR Detection kit (Sigma Aldrich, USA).

Morphological assessment was performed using Nikon Eclipse Ti phase-contrast, fluorescence microscope, and NIS-Elements Imaging Processing software (Nikon, Tokyo, Japan).

Evaluation of cell viability in the conditioned medium allows for a dynamic analysis of the interaction of the tested material with the culture medium imitating body fluids. This method allows to predict how the test material will interact on a longer contact with body fluids and how it will affect the biological response.

Tested titanium discs were placed separately in 24-well culture plate and covered with 1 ml of supplemented medium. The plate was placed on an orbital shaker and incubated continuously at 37°C at 300 rpm for 24, 72, and 168 hours. The appropriate medium incubated under analogous conditions but without titanium insert was used as a control.

The cells were seeded into 96-well plates at 2 *∗* 10^4^ cells/well BJ. After 24 h, when the confluency reached 80%, the culture medium was aspirated and the cells were covered with conditioned medium and incubated for next 24 hours. Cell treated with 1% Triton *X*-100 were used as the positive control.

#### 2.3.2. MTT Test

The viability of the cells was examined by the standard MTT assay, using the MTT Cell Proliferation Assay kit (Invitrogen, US). In this test, the activity of mitochondrial enzyme-succinate dehydrogenase is used. This enzyme in living cells is responsible for the transformation of soluble tetrazolium salt 3-(4,5-dimethylthiazol-2-yl)-2,5-diphenyltetrazolium bromide) to water-insoluble purple formazan crystals. Following 4 h incubation, the medium with MTT was removed, and the formed crystals were dissolved in DMSO. The solution absorbency was measured at 540 nm, using the PowerWave™ microplate spectrophotometer (Bio-Tek Instruments, USA). The experiment was repeated 3 times, and the measurements were performed in triplicate.

The results were analysed statistically in the STATISTICA vs. 13 application (StaftSoft, Poland). Data were calculated as mean ± SD. To compare more than two groups, the one-way analysis of variance ANOVA and post hoc multiple comparisons on a basis of Tukey's HSD test were used. All parameters were considered statistically significantly different if *p* values were less than 0.05.

#### 2.3.3. Cell Growth Evaluation in Direct Contact with the Examined Disks

The cells were seeded onto a 12-well plate, where a titanium insert was placed in each well. The cultures were incubated in standard conditions, and cell growth near inserts was observed for 3 days. For better visualization of cells and accurate counting, they were stained with Hoechst 33342. Staining solution was prepared by diluting stock solution (10 mg/ml) at a ratio of 1 : 2000 in the fresh medium. This dye penetrates through intact cell membranes and binds to dsDNA. Then, it is excited by ultraviolet light, and the stained nuclei can be observed by using a fluorescence microscope. In the study, the number of stained cells nuclei was measured on a specific surface of culture plate (89000 *μ*m^2^) nearest to the insert.

## 3. Results and Discussion

### 3.1. Morphology and Geometrical Structure of Surface

**Figure 2:**
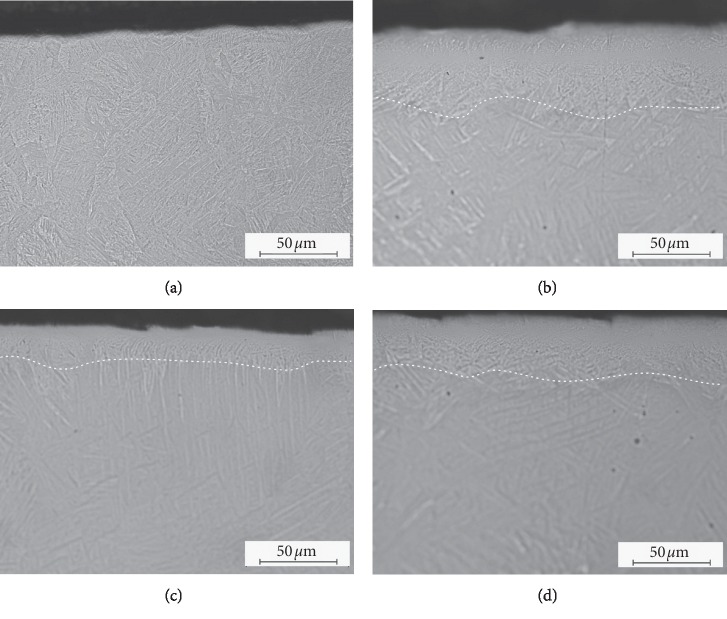
The cross section of specimens showing modified surface layer after shot peening using the pressure of 0.4 MPa: (a) unmodified surface after DMLS (reference sample); (b) shot made of CrNi steel; (c) nutshell granules; (d) ceramic beads.

In the course of metallographic observations of specimen cross section, it has been observed that plastic deformations after shot peening caused significant changes near the surface (Figure 2). Significant difference has been observed in the grain size of the surface layer and the bulk substrate. Significant grain size reduction is visible in the surface layer (Figure 2) marked with broken lines. The changes in microstructure reach the depth of about 60 *μ*m in the case of substrates subjected to the shot peening process by means of ceramic balls and about 25/30 *μ*m in the case of surfaces modified by means of nut shells.

The changes in the surface layer associated with grain size reduction have been also observed by Ahmed et al. [11] and Dai et al. [12] at different shot peening parameters. Furthermore, Kameyama and Komotori [13] observed that microstructure near the surface may additionally exhibit lamellar features associated with the transfer of shot particles fragments. In their study, they presented a model of local lamellar microstructure creation under the influence of fine particle peening.

Sa parameter—arithmetic average of surface roughness—used for the evaluation of surface development. According to the available literature [6, 11, 14], Sa parameter is the most representative parameters for surface evaluation after the shot peening process. The results obtained from profilometric measurements (Table 2) demonstrated that the values of the Sa parameter were the lowest in the case of surfaces processed by means of CrNi shot and ceramic balls. At the same time, the differences in the roughness value between the both surfaces are relatively low (not statistically significant). It is worth noting that the diameter of CrNi shot was almost three times greater than the diameter of ceramic balls. Larger size of grain causes the creation of larger indentations in the surface layer which translates into reduced number of indentations per surface area unit. However, surface processing by means of sharp-edged nut shells caused the highest increase of the Sa parameter. Owing to their shape, sharp-edged nut shells are more easily located (driven) in surface layer of titanium alloy, additionally increasing surface roughness [3] and simultaneously causing microstructure changes at relatively smallest depth (Figure 2(c)). In accordance with the studies of Lee et al. [10], a greater increase of surface development contributes to better osteointegration of implants, while osteoblasts proliferation remains greater on smooth titanium. However, Tuomi et al. [5] indicate that, although the higher roughness of implants obtained from 3D printing contributes to better adhesion of tissues to metal biomaterials, such types of surfaces, in the case of dynamically loaded implants, may constitute the areas with reduced fatigue life.

### 3.2. Mechanical Properties

**Figure 3:**
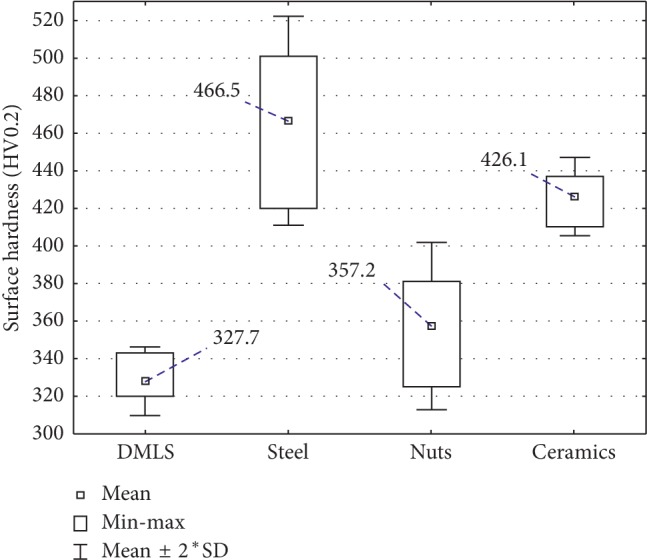
The variation of surface hardness of Ti-6Al-4V alloy after treatment shot peening.

The hardness testing (Figure 3) demonstrated the increase of average hardness values for all the treated surfaces in comparison with specimens obtained directly after DMSL sintering. The highest reinforcement of surface layer has been achieved in the case of surface subjected to steel shot peening and then by means of nut shells which translated into hardness increase by about ∼42% and ∼30% correspondingly, in relation to the reference sample. In this regard, the surface subjected to processing by means of nut shells was the worst-case condition, and its hardness was slightly higher in comparison with reference sample (DMLS). The processing by means of nut shells caused only the hardness increase by about ∼9% in relation to the reference sample. Average hardness of the reference hardness surface was equal to 327.7HV, i.e., it was almost similar to the value declared by the manufacturer: EOS GmbH–320HV.

In order to verify whether the achieved changes are statistically significant, the analysis has been carried out by means of STATISTICA vs. 13 program using parametric tests for independent tests. On the grounds of a statistical analysis by means of the Shapiro–Wilk test for surface hardness measurement, it has been demonstrated that obtained results do not have a normal distribution, *p* < 0.05 (assuming *α* = 0.05). Therefore, *p* < *α* and there are grounds for rejection of the hypothesis of normal distribution for the feature being tested. Therefore, the nonparametric tests for Kruskal–Wallis independent tests (for *α* = 0.05) have been applied for further statistical analysis. On the grounds of statistical analysis, it has been demonstrated that the differences in reinforcement of surface layer are statistically significant (*p* < 0.05) only between reference surface (DMLS) and modified by means of steel shot and ceramic balls as well as between surface modified by means of steel shot and nutshells.

Hardness increase for surfaces subjected to the shot peening process results from plastic deformations of surface layer and is associated with the effect of hard grains striking towards the component surface. According to the publications available, a hard nanocrystalline layer is created on the surface of parts being processed and causes the increase of hardness and corrosion resistance [13, 15].

**Figure 4:**
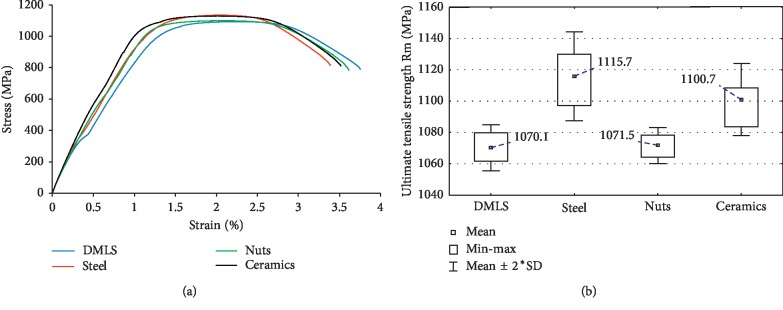
Tensile test: (a) stress-strain curves of various shot peening treated specimens and (b) box and whiskers plots of ultimate tensile strength.

Tensile strength tests (Figure 4) demonstrated similar correlations between the groups of specimens to those obtained from hardness tests. Therefore, the most favourable values of Rm have been obtained for surfaces processed by means of ceramic balls and steel shot. Such modification of surface layer indicated an average increase of tensile strength by 30 and 45 MPa correspondingly, in relation to an unmodified surface, i.e., about 3.7 and 4.2%. However, the treatment by means of nut shells did not demonstrate any statistically significant differences in relation to an unmodified surface. Such effect results from weak reinforcement of surface layer at an achieved level of relatively higher roughness. Insufficient surface quality translates into strength.

Presumably, the results for surfaces subjected to shot peening achieved in fatigue tests could be more favourable than those achieved in static tensile tests. In the opinion of Benedetti et al. [6], the surface hardening by means of shot introduces favourable compressive stresses and reduces porosity of surface layers significantly contributing to the increase of fatigue strength. However, Ganesh et al. [14] indicate that it is possible to achieve a significant improvement of tensile strength through proper choice of technological parameters for the shot peening process. In the case of products from 3D printing, except for the condition of surface layer, mainly the structural discontinuities in the form pores in the product itself are of key importance for the strength. Thijs et al. [16] emphasize that depending on applied technological printing processes, gas bubbles can be created in the material. Mierzejewska et al. [17] indicate certain imperfections of the DMLS process. In the course of analysis of specimens cracking surface in strength tests, they have observed voids and unmelted powder (attributed to entrapped gas in the melt pool and lack of melting during fabrication).

Furthermore, Konečná et al. [18] found that the crack initiation in fractures after tensile tests (for three different orientations of specimens) mainly depends on specimen surface quality. The crack initiation locations occurred in the form of unmelted or partially melted metal powder particles deposited on the specimen surface, particularly in the case of specimens with *Y*-*Z* and *Z*-*X* orientations.

**Figure 5:**
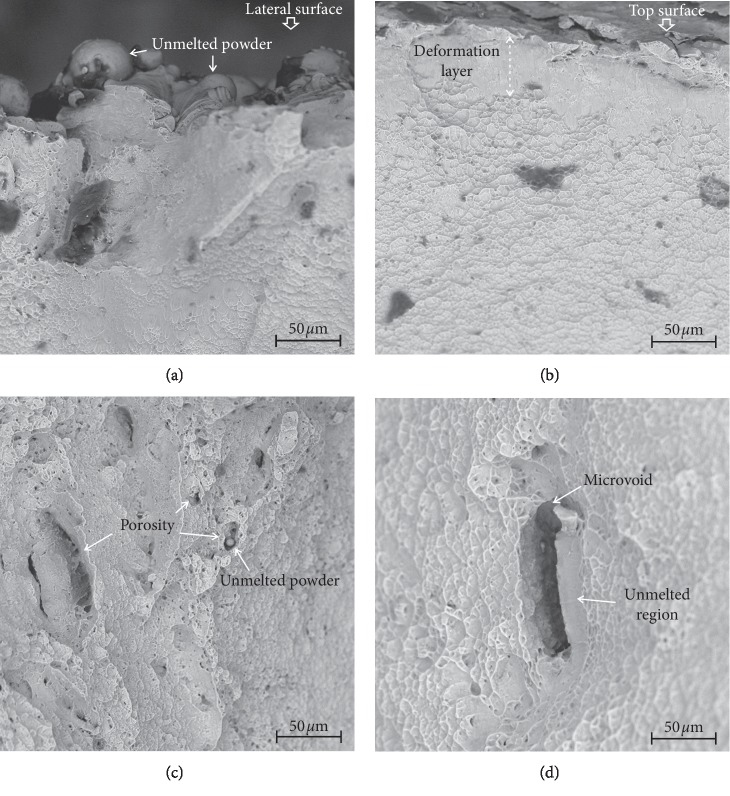
Fracture surface (a) of unmodified sample (reference), (b) after shot peening using ceramics beads, and (c-d) of sample with same imperfections of the DMLS process.

The findings e in [17, 18] have confirmed fractographic analysis of cracking surface after strength tests (Figure 5). In all cases, ductile fractures have been observed. Fracture morphology resembles honeycombs with many small voids. There are no significant differences between specimens cracking surfaces subjected to processing by means of different types of shots except for the observed superficial layer resulting from material strengthening after shot peening. However, the defects in the form of limited pores and unmelted metal powder particles constituted the principal crack initiation locations in all the cases which caused stress concentration for crack growth.

### 3.3. Biological Properties

**Figure 6:**
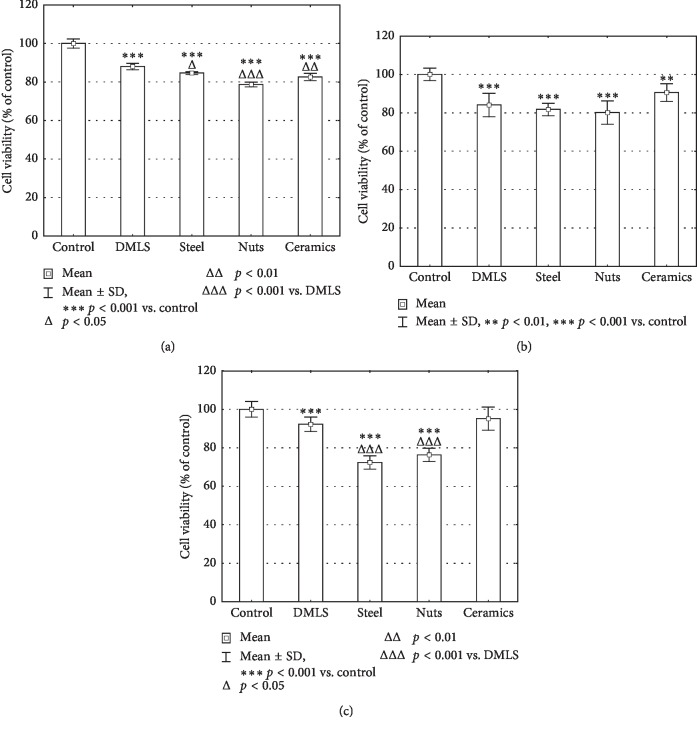
MTT test results for BJ cells treated with tested titanium discs extracts according to kind of disc modification for: (a) 24 h, (b) 72 h, and (c) 168 h of extraction. Data are expressed as % of values obtained for control.

MTT test results (Figure 6) revealed that all 24 h-conditioned media revealed statistically significant toxicity against tested cells as compared with the control cells (84.67 ± 0.75, 78, 73 ± 1.28, and 82.56 ± 1.84% for surfaces modified by means of steel shot, nut shells, and ceramic balls, respectively) as well as compared with cells treated with medium conditioned with the DMLS insert (88.06 ± 1.73%). A significant decrease in cell viability was observed in cultures incubated in media conditioned for 72 h with all inserts (81.74 ± 3.28, 80.07 ± 6.04, and 90.57 ± 4.66% for steel shot, nut shells, and ceramic balls, respectively); however, there were no significant differences between DMLS (84.06 ± 6.10%) and other inserts. Among long-conditioned media (168 h), only the surfaces modified by means of steel shot and nut shells revealed toxicity (as compared to DMLS)—72.40 ± 3.47 and 76.36 ± 3.50% of cells viability. The results show that the insert modified by means of ceramic balls extract exerts the weakest effects on cells, and the viability of cells cultured in 168 h conditioned media was similar to that in the cells incubated in the control medium (without conditioning)—95.15 ± 6.04%.

**Figure 7:**
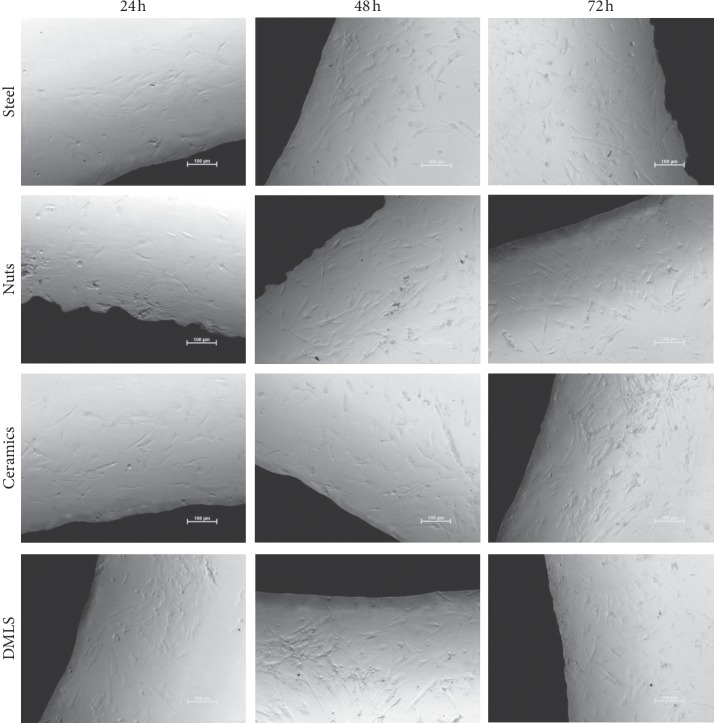
Cell growth and proliferation in direct contact with the examined titanium inserts. The area displayed in black belongs to the specimen made of titanium.

**Figure 8:**
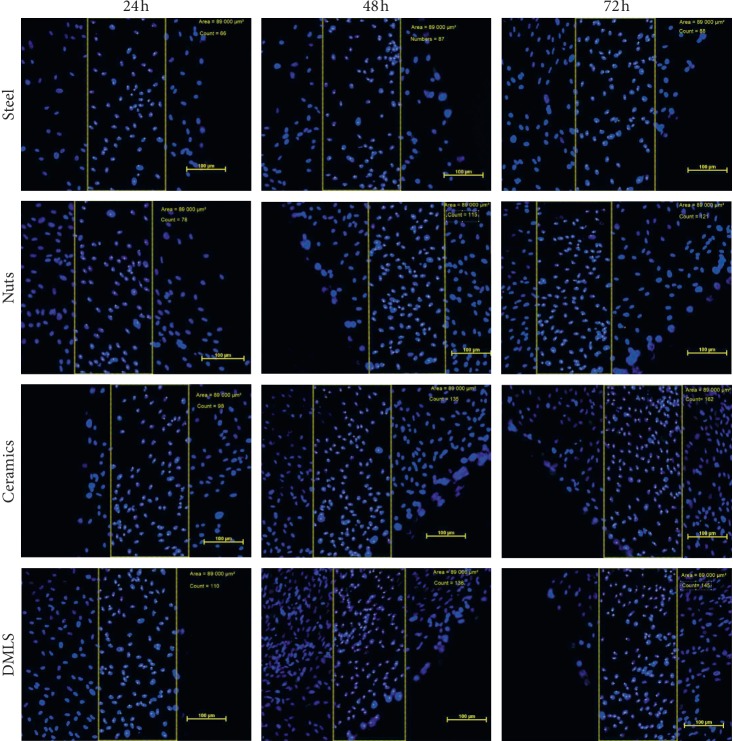
Selected microphotography after analysis (staining the nuclei with fluorescent dye).

There were no morphological differences between cells growing in the presence of the tested inserts (Figure 7). There was also no inhibition of cell proliferation near the edge of the inserts. However, after staining the nuclei with fluorescent dye and counting them in a certain area of the field of view after 24, 48, and 72 h, it turned out that the cells growing near the modified by means of steel shot inserts have the weakest proliferation potential (Figure 8). In contrast to the other cultures, the cells incubated with insert modified by means of ceramic balls after 72 hours of experiments still intensively proliferated.

The biological tests demonstrated that the surfaces subjected to the treatment by means of ceramic balls are characterized by the highest biocompatibility. High biocompatibility of such surfaces results from bioinertness of ceramic material and from low roughness levels of surfaces subjected to the treatment by the means of ceramic balls. The surfaces subjected to the treatment by the means of CrNi steel shot were characterized by the poorest parameters although the surface roughness after such kind of treatment was low. Such a condition results from the cancerogenic properties of elements, i.e., Cr or Ni, which are located (driven) in the surface layer after the shot peening process [3, 7, 19]. Therefore, owing to favourable properties obtained in mechanical tests of specimens subjected to shot peening process by means of steel shot, such a kind of treatment will better prove itself in the case of fabrication of medical instruments than in the case of implant surface modification. In spite of their worse strength parameters, the specimens subjected to the shot peening process by the means of nut shells are slightly better with regard to cytotoxicity in comparison with the surfaces subjected to the shot peening process by means of steel shot. Nevertheless, any information about cytotoxicity of surfaces subjected to the shot peening process by means of an organic medium is still lacking in the literature.

From biocompatibility tests it appears, in an indirect manner, that the influence of differences in roughness on biocompatibility is not as prevailing as the influence of the type of the shot applied. Tuomi et al. [5] found a high viability of L929 cells after the incubation period of 24 h in the extract for unprocessed and polished specimens after 3D printing. Similarly, the studies carried out by Vaithilingam et al. [20] evaluating cytotoxicity of 3T3 cells on surfaces polished and obtained from 3D printing have not revealed any significant differences. Seven-day tests demonstrated that the cells were viable on the both surfaces and it has been found that any metal ions from Ti-6Al-4V alloy have not been dissolved in the culture medium. Furthermore, favourable properties of specimens obtained from 3D printing and subjected to the shot peening process are indicated by Benedetti et al. [6]. It has been demonstrated that the titanium specimens with various surface roughness (after electropolishing and shot peening process) are characterized by quite similar favourable behaviour at different times with regard to cell growth and proliferation.

## 4. Conclusion

The following conclusions have been drawn from the tests carried out by authors:

- The shot peening process carried out by the means of steel shot as well as by the means of ceramic ball-shot caused the reduction of roughness in relation to the untreated-reference DMLS manufactured surface. However, in the case of treatment by means of nut shells, increased roughness has been observed in comparison with the reference surface. From biocompatibility tests, it appears that the influence of differences in roughness on biocompatibility is not as prevailing as the type of applied peening shot-type.
- The shot peening process carried out by the means of steel shot as well as by means of ceramic balls causes an increase in the of the modified surface. At the same time, statistically significant and most favourable effects have been obtained for surfaces modified by the means of steel shot as well as ceramic balls (hardness increases by about ∼42% and ∼30% correspondingly vs. reference surface).
- The static tensile test and fractographic analysis demonstrated structural imperfections of DMLS manufactured components. Insignificant increase in the ultimate tensile strength Rm (about 4%) has been obtained for surfaces modified by means of steel shot and ceramic balls. In all cases, the places of crack initiation occurred in the form of *limited pores and* unmelted metal powder particles.
- The lowest cytotoxicity has been found in the case of surfaces modified correspondingly by means of ceramic balls < nut shells < steel shot. Furthermore, the cells growing in vicinity of specimen-modified means of ceramic balls were characterized by the highest proliferation potential.

Generally, the tests demonstrated that mechanical properties were most favourable in the case of surfaces modified with steel shot. However, with regard to biocompatibility, the processing by the means of ceramic balls is the most preferred method in the context of processing of implants obtained from 3D printing.

## Figures and Tables

**Table 1:** Parameters of shot for shot peening.

Shot	Typical chemical composition (%)		Average grain size (*μ*m)	Grain shape	Hardness
Stainless steel shot – CrNi	Cr	16–20	400–900	Spherical	235 HV
	Ni	7–9			
	Si	1.8–2.2			
	Mn	0.7–1.2			
	C	0.05–0.2			
	Fe	Bal.			

Nutshell granules	Nonferrous, organic blasting media		450–800	Angular	Approx. 2.5–3.5 mohs

Ceramic beads	ZrO_2_	61.98	125–250	Spherical	Approx. 7–7.5 mohs
	SiO_2_	27.77			
	Al_2_O_3_	4.57			
	CaO	3.47			
	TiO_2_	0.34			
	Fe_2_O_3_	0.14			

**Table 2:** Summary of average arithmetical deviation of roughness Sa (*μ*m) for surfaces being tested.

Roughness of shot peened surfaces	Steel CrNi	Nutshell granules	Ceramics	Unmodified surface after DMLS
Sa (*μ*m)	6.864	7.808	6.893	7.433

## Data Availability

The data used to support the findings of this study are available from the corresponding author upon request.

## References

[1] Aimar A., Palermo A., Innocenti B. (2019). The role of 3D printing in medical applications: a state of the art. *Journal of Healthcare Engineering*.

[2] Haruna W. S. W., Manam N. S., Kamariah M. S. I. N. (2018). A review of powdered additive manufacturing techniques for Ti-6Al-4V biomedical applications. *Powder Technology*.

[3] Żebrowski R., Walczak M. (2019). Effect of the shop peening on surface properties and tribological performance of Ti-6Al-4V alloy produced by means of DMLS technology. *Archives of Metallurgy and Materials*.

[4] Garcia J., Yang Z., Mongrain R., Leask R. L., Lachapelle K. (2018). 3D printing materials and their use in medicaleducation: a review of current technology and trends for the future. *BMJ Simulation and Technology Enhanced Learning*.

[5] Tuomi J. T., Björkstrand R. V., Pernu M. L. (2017). In vitro cytotoxicity and surface topography evaluation of additive manufacturing titanium implant materials. *Journal of Material Science: Materials in Medicine*.

[6] Benedetti M., Torresani E., Leoni M. (2017). The effect of post-sintering treatments on the fatigue and biological behavior of Ti-6Al-4V ELI parts made by selective laser melting. *Journal of the Mechanical Behavior of Biomedical Materials*.

[7] Żebrowski R., Walczak M., Klepka T., Pasierbiewicz K. (2019). Effect of the shot peening on surface properties of Ti-6Al-4V alloy produced by means of DMLS technology. *Eksploatacja I Niezawodnosc—Maintenance and Reliability*.

[8] Havlikova J., Strasky J., Vandrovcova M. (2014). Innovative surface modification of Ti-6Al-4V alloy with a positive effect on osteoblast proliferation and fatigue performance. *Materials Science and Engineering C—Materials for Biological Applications*.

[9] Velasco-Ortega E., Alfonso-Rodríguez C. A., Monsalve-Guil L. (2016). Relevant aspects in the surface properties in titanium dental implants for the cellular viability. *Materials Science and Engineering C—Materials for Biological Applications*.

[10] Lee E. M., Smith K., Gall K., Boyan B. D., Schwartz Z. (2016). Change in surface roughness by dynamic shape-memory acrylate networks enhances osteoblast differentiation. *Biomaterials*.

[11] Ahmed A. A., Mhaede M., Wollmann M., Wagner L. (2016). Effect of micro shot peening on the mechanical properties and corrosion behavior of two microstructure Ti-6Al-4V alloy. *Applied Surface Science*.

[12] Dai S., Zhu Y., Huang Z. (2016). Microstructure evolution and strengthening mechanisms of pure titanium with nano-structured surface obtained by high energy shot peening. *Vacuum*.

[13] Kameyama Y., Komotori J. (2009). Effect of micro ploughing during fine particle peening process on the microstructure of metallic materials. *Journal of Materials Processing Technology*.

[14] Ganesh B. K. C., Sha W., Ramanaiah N., Krishnaiah A. (2014). Effect of shotpeening on sliding wear and tensile behavior of titanium implant alloys. *Materials and Design*.

[15] Jelliti S., Richard C., Retraint D., Roland T., Chemkhi M., Demangel C. (2013). Effect of surface nanocrystallization on the corrosion behavior of Ti-6Al-4V titanium alloy. *Surface and Coatings Technolology*.

[16] Thijs L., Kempen K., Kruth J.-P., Van Humbeeck J. (2013). Fine-structured aluminium products with controllable texture by selective laser melting of pre-alloyed AlSi10Mg powder. *Acta Materialia*.

[17] Mierzejewska Ż.A., Hudák R., Sidun J. (2019). Mechanical properties and microstructure of DMLS Ti6Al4V alloy dedicated to biomedical application. *Materials*.

[18] Konečná R., Nicoletto G., Bača A., Kunz L. (2016). High cycle fatigue life of Ti6Al4V alloy produced by direct metal laser sintering. *Solid State Phenomena*.

[19] Al-Hiyasat A. S., Darmani H. (2005). The effects of recasting on the cytotoxicity of base metal alloys. *The Journal of Prosthetic Dentistry*.

[20] Vaithilingam J., Prina E., Goodridge R. D. (2016). Surface chemistry of Ti6Al4V components fabricated using selective laser melting for biomedical applications. *Materials Science and Engineering C—Materials for Biological Applications*.

